# Alzheimer's Disease and Stroke: A Tangled Neurological Conundrum

**DOI:** 10.7759/cureus.25005

**Published:** 2022-05-15

**Authors:** Anuradha Gupta, Kamsika Uthayaseelan, Kivonika Uthayaseelan, Monika Kadari, Muhammad Subhan, Nisha Saji Parel, Parimi vamsi Krishna, Ibrahim Sange

**Affiliations:** 1 Research, Government Medical College, Nagpur, IND; 2 Internal Medicine, All Saints University College of Medicine, Saint Vincent and the Grenadines, Kingstown, VCT; 3 Internal Medicine, All Saints University School of Medicine, Roseau, DMA; 4 Internal Medicine, Bhaskar Medical College, Hyderabad, IND; 5 Internal Medicine, Allama Iqbal Medical College, Lahore, PAK; 6 Family Medicine, Tbilisi State Medical University, Tbilisi, GEO; 7 Internal Medicine, JJM Medical College, Davanagere, IND; 8 Research, K. J. Somaiya Medical College and Research Center, Mumbai, IND

**Keywords:** memantine, acetylcholinesterase inhibitors, apolipoprotein, cerebral amyloid angiopathy (caa), cerebrovascular disorders, hemorrhagic stroke, ischemic stroke, stroke, alzheimer's disease, alzheimer’s dementia

## Abstract

A neurodegenerative disorder, Alzheimer's disease (AD), is characterized by dementia in which there is an age-related decline in cognition and higher functions. Stroke is a cerebrovascular disorder that frequently presents in old age and is a known risk factor for AD development. However, the association that AD can be a risk factor for stroke is not well-studied. This review article compiled various studies that pointed out the association between stroke development in patients with dementia, particularly AD-related dementia. The pathophysiological progression of stroke in AD cases and the genetic makeup possibly affecting the interrelation between these disorders were analyzed in detail using currently available data and studies. Therapeutic and management modalities already in use for AD were put together, and the possibility of early intervention in such patients benefitting cerebrovascular pathologies, particularly stroke-related, was explored. Prognostic differences between patients of stroke with and without AD were also reviewed, and how appropriate management can reduce the burden on health care settings when both present simultaneously was emphasized.

## Introduction and background

An irreversible neurodegenerative disorder, Alzheimer's Disease (AD) is characterized by dementia in which there is an age-related deterioration of cognition and higher functions [1]. AD was first described in 1906 by Alois Alzheimer, who identified a patient Auguste D with symptoms of psychosis and deficits in memory, social skills, and cognition [2]. AD dementia has been defined by a specific pattern of deterioration in cognition and function accompanied by discrete neuropathology and aging [1]. It has been estimated that currently, 24.3 million people have dementia, and this number will go as high as 81.9 million by 2040 [2]. Studying the proteins present in cerebrospinal fluid (CSF) by performing dual clustering technique non-negative matrix factorization, three subtypes of AD have been defined - subtype 1, subtype 2, and subtype 3 [3]. Risk factors for AD are categorized as non-modifiable (old age, female sex, family history, genetics), modifiable (smoking, illiteracy, vascular disorders, head trauma, heart diseases, cerebrovascular disorders, inflammation, infections), and clinical (extrapyramidal signs, initial level of cognition) [4, 5]. Pathogenesis of AD includes the formation of neuritic plaques (NP) and neurofibrillary tangles (NFT) as a result of Aβ amyloid accumulation, mainly in the medial temporal lobe and neocortical structures [6]. Tau is a microtubule-associated protein in the brain that undergoes hyperphosphorylation to form NFTs leading to brain atrophy, synaptic loss, neurotoxicity, and finally, low cognition [7, 8]. This disease has shown an association with mutations in amyloid precursor protein (APP), presenilin 1 (PSEN1), presenilin 2 (PSEN2), apolipoprotein E (ApoE), and a disintegrin and metalloprotease 10 (ADAM10) [9]. The clinical symptoms of AD differ from patient to patient and comprise progressive derangements in memory, language, thinking, and personality, ultimately leading to the inability to perform daily living activities [10]. Although the diagnosis is predominantly clinical, it can be confirmed by autopsy analysis of brain tissue [11].

In contrast, diagnostic modalities in living cases include magnetic resonance imaging (MRI), positron emission tomography (PET) scan, as well as pathophysiological biomarkers like CSF analysis for Aβ or tau and amyloid PET intake [12]. Lifestyle modifications like a healthy diet, smoking cessation, and exercise together with pharmacotherapy using acetylcholinesterase inhibitors (AChEI) like donepezil, rivastigmine, and galantamine, as well as N-methyl d-aspartate (NMDA) antagonists like memantine, are currently used for the management of AD [4]. Today, therapies using disease-modifying drugs targeting the Aβ pathway, tau pathology, and chaperon proteins are being used in advanced settings [4]. According to a systematic review and meta-analysis performed by Zhou J et al. in 2015, stroke has been defined as a known risk factor for AD development [13]. However, the exact opposite association that AD increases the chances of developing stroke has not been emphasized much in the available data and studies [13]. This article aims to explore the pathophysiological progression of stroke in AD patients and identify the differences in prognosis between patients with and without AD.

## Review

Etiology and pathophysiology

**Figure 1:**
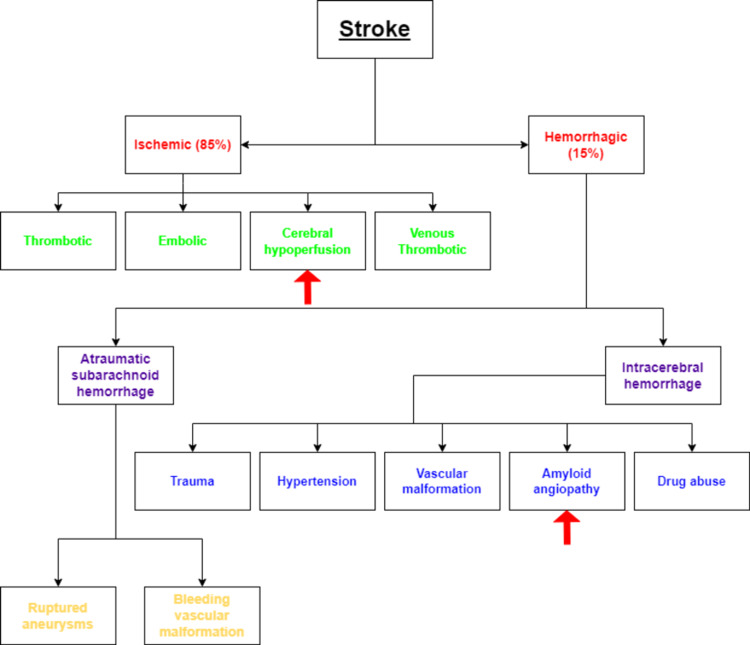
Etiological classification of stroke Red arrows point out the causes of stroke connected to Alzheimer's disease Image credit: Anuradha Gupta

**Figure 2:**
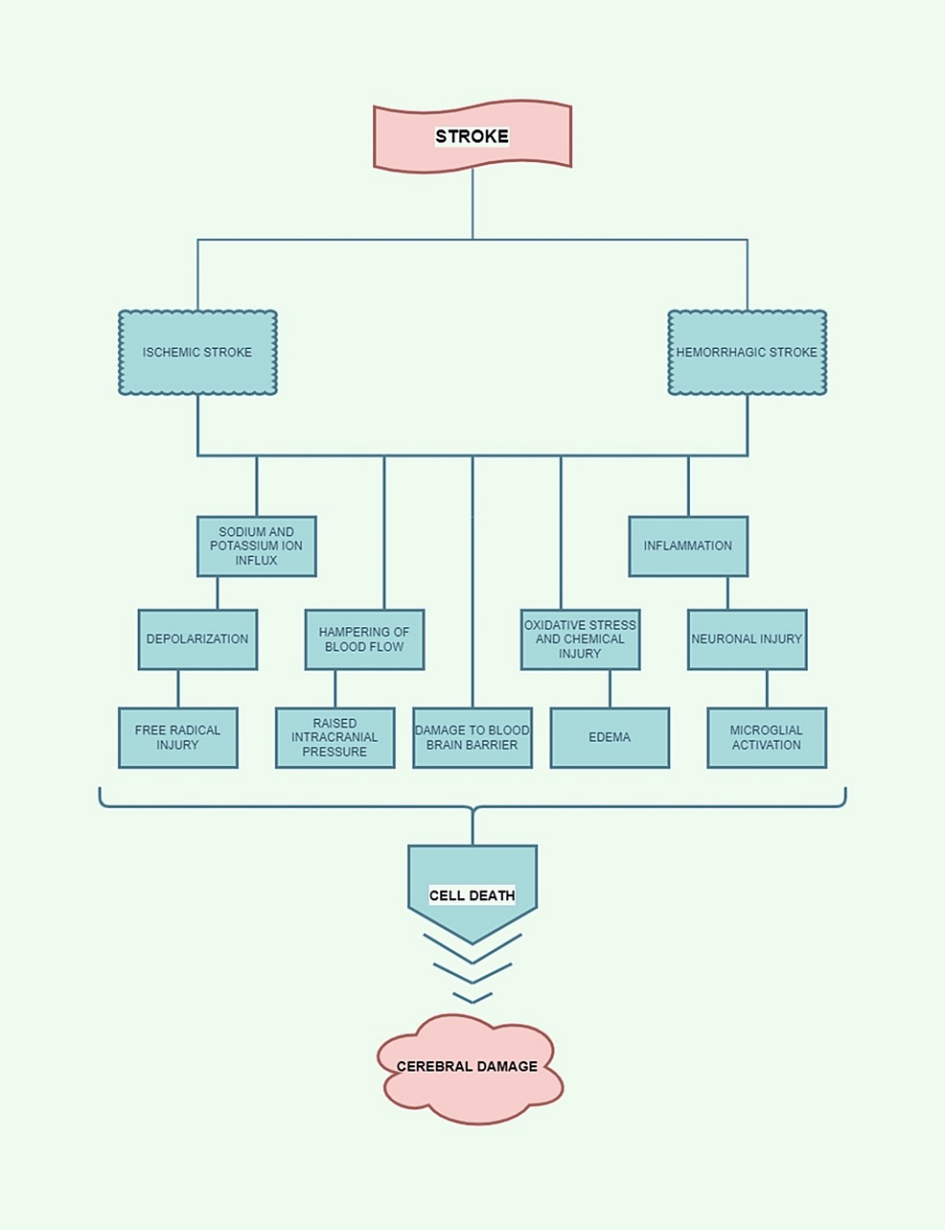
Pathophysiology of stroke Image credit: Anuradha Gupta

Stroke ranks as the second most common cause of death and disability in the entire world [14]. Low- to middle-income countries witnessed a doubled incidence of stroke between 1990 and 2016 [14]. However, in high-income countries, it decreased by 42% over the same period [14]. At present, stroke rates are highest in developing countries [14]. Stroke is a cerebrovascular disorder caused by a halt in the flow within the brain's blood vessels followed by clot formation [14]. The result is a blockage of the arteries and the breakage of the damaged blood vessels, resulting in intracerebral (IC) bleeds [14]. Clinically stroke is broadly divided into two types, ischemic stroke (IS) (85% of the cases) owing to the lack of blood supply and oxygen to brain parenchyma; hemorrhagic stroke (HS) (15% of the cases) manifesting as a result of leaky or damaged blood vessels (Figure 1, 2) [14]. IS has been further classified into four subtypes depending on the etiology as thrombotic (large vessel and small vessel type), embolic, cerebral hypo-perfusion (watershed region stroke), and venous thrombosis [15]. On the other hand, HS is categorized into atraumatic subarachnoid hemorrhage (SAH) due to ruptured aneurysms or bleeding vascular malformations, and IC hemorrhage is caused by factors like trauma, hypertension, specific vascular malformations, amyloid angiopathy as well as drug abuse (Figure 1) [15].

The stroke pathophysiology has a labyrinthine course that entails neuroprotection, oxidative injury, apoptosis, necrosis, inflammation, excitotoxicity, and angiogenesis [15]. It also encompasses processes like Aβ amyloid protein metabolism, oxidative injury, hormonal pathways, and defects in adrenergic, serotonergic, glutamatergic, and dopaminergic pathways [16]. The brain is particularly susceptible to the detrimental effects of ischemia because it has a low respiratory reserve and relies exclusively on aerobic metabolism for its energy requirements [15]. Due to collateral circulation, a gamut of severity related to damage to brain parenchyma is observed in IS ranging from a complete loss of activity due to cell death in some regions to partially damaged tissues which retain the ability to recover with proper management [15]. On the other hand, HS occurs due to breakage in blood vessels and can cause damage in the wake of hypoxia, an irritant effect on the brain due to spilled blood, and raised intracranial pressure making HS a bigger villain than the IS in the prognosis of the disease as a whole [15]. Stroke and AD are associated because cerebrovascular disorders such as stroke (both ischemic and hemorrhagic types) are among the well-known risk factors for developing AD [4, 5]. On the other hand, AD has also been established as a potential risk factor for stroke development, but this idea has not been studied much [13].

The pathological hallmarks of AD are the NPs and the NFTs [6]. NPs are formed due to the buildup of Aβ amyloid peptides formed because of disturbance in proteolytic cleavage of APPs [6]. In contrast, NFTs are formed due to changes in the cytoskeleton because of microtubule-associated hyperphosphorylated tau protein [6]. Aβ peptide aggregates into insoluble oligomers and proto-fibrils that form senile plaques or NPs, later accumulating into the brain's extracellular spaces [6]. This accumulation ultimately activates a neurotoxic cascade, leading to cytoskeletal changes, neuronal dysfunction, and cell death in the end [6]. Recent clinical data has pointed out that more than 30% of AD patients have some concurrent cerebrovascular pathology involving the cells present within the blood-brain barrier (BBB) [17]. The accumulated amyloid-β damages larger perforating arteries and the cerebral capillaries, which form the BBB [17]. This notion is further supported by a study performed by Keaney et al. in 2015, which suggested that the clearance of already accumulated Aβ amyloid in AD is severely hampered because of tight junction proteins in the BBB [18]. The cerebrovascular pathologies stated above include cerebral amyloid angiopathy (CAA), degenerative microangiopathy, cerebral infarcts, and IC hemorrhages (Table 1) [17].

**Table 1:** Percentage-wise estimate of various cerebrovascular pathologies in Alzheimer's disease CAA, cerebral amyloid angiopathy; ApoE, apolipoprotein E; AD, Alzheimer's disease; IC, intracerebral

Sr. no.	VASCULAR LESIONS	PERCENTAGE	SPECIFIC FEATURE OR MARKER
1.	Cerebral micro-vessels degeneration	100%	CD34, GLUT1, basement membrane thickening, collagen IV, loss of endothelial markers
2.	CAA	98%	Inflammatory markers, Aβ peptides, Cystatin C protein
3.	Localization of serum proteins	80%	ApoE in AD lesions, complement, P component
4.	Large cerebral infarcts and cortical micro-infarcts	36%	Variably distributed and sized
5.	Diffuse white matter disease	35%	Periventricular and deep white matter lesions
6.	Presence of lobar and IC hemorrhages	10%	CAA-related IC hemorrhages

According to a study performed by Charidimou et al. in 2017, CAA is a part of protein elimination failure angiopathy, which worsens the Aβ accumulation, activates vascular injury pathways, and impairs vascular physiology [19]. This idea can be compared along the lines of another study performed in 2018 by Iadecola C and Gottesman RF, which inferred that accumulated Aβ could reduce cerebral blood flow and cause neurovascular dysfunction [20]. This type of neurovascular dysfunction eventually increases the chances of ischemia in the affected brain [20]. Tolppanen et al. conducted a register-based matched cohort study in Finland for a brief period of three years on community-dwelling people with the clinical diagnosis of AD to know about the difference between IS and HS incidence in people with and without AD [21]. After the follow-up, it was determined that younger patients with the clinical diagnosis of AD have a higher chance of developing HS than people who do not suffer from AD [21]. This finding can be compared and contrasted with another case-control matched analysis done by Chi et al. in 2013 for ten years using Taiwan's National Health Insurance Research Database on people with a diagnosis of AD [22]. The results showed a considerably increased IS and IC hemorrhage risk in AD cases [22].

It is known that the APOE gene is one of the most critical risk factors for the development of AD [23]. The APOE gene codes for a glycoprotein ApoE, which is highly expressed in the brain, and has three main isoforms - ApoE2, ApoE3, and ApoE4 [23]. The ApoE4 isoform has been associated with the accumulation of NPs and NFTs in AD [23]. A study carried out by McCarron MO and Nicoll JA in 2000 suggested that Aβ proteins are deposited in the cerebral blood vessel walls due to the epsilon4+ genotype [24]. In contrast, a potential risk factor of hemorrhage in amyloid-laden blood vessels is the epsilon2+ genotype [24]. Similar results were obtained in a population-based study conducted later in 2002 by Woo et al. with 188 HS cases and 366 control subjects in the Greater Cincinnati/Northern Kentucky region [25]. It was concluded that about one-third of the patients of lobar IC hemorrhage possess an ApoE4 or ApoE2 allele, and nearly half of all the cases of non-lobar IC hemorrhage are attributable to hypertension [25]. Similarly, systematic review and meta-analyses were performed by Sudlow et al. using 31 eligible studies to know about the risk associated with the presence of different alleles of APOE genotype on the IS and the HS [26]. This study suggested that epsilon4+ genotypes were significantly associated with IS and SAH and non-significantly with IC hemorrhage, whereas epsilon2+ genotypes were associated with only IC hemorrhage [26]. A study by Fekih-Mrissa et al. in the Tunisian population established an association between APO ɛ4 allele with concomitant AD and stroke [27]. Therefore, it can be said that the pathophysiology of IC hemorrhage varies by location, and it is significantly associated with the APOE genotype, which has a very well-known association with the pathophysiology of AD [23-26]. Olichney et al. performed a retrospective clinicopathological study of 145 autopsy-confirmed AD cases in 1995. They suggested that severe amyloid angiopathy and hypertension in AD patients produce multiplicative injuries to the neuronal vasculature and increase the frequency of cerebral infarction [28]. Later in 2000, Olichney et al. again tried to test the association of CAA with cerebrovascular events and the APOE4 genotype after reviewing 306 autopsy-confirmed AD cases from the University of California, San Diego [29]. It was determined that advanced cases of CAA were associated with an increased risk of cerebrovascular events irrespective of APOE4 genotype [29]. In the same spirit, a systematic review was performed by Khan et al. in 2013 using pooled data from 41 studies [30]. They concluded that the APOE genotype shows a positive dose-response association with IS [30]. There still is a need for further evaluation into the pathways and mechanisms through which vascular lesions are developed due to CAA's presence in AD cases [30]. Studies establishing the association between concomitant AD and stroke, as well as their link with APOE genotype have been compiled in Table 2.

**Table 2:** Studies establishing the association between concomitant AD and stroke, as well as their link with APOE genotype AD, Alzheimer's disease; HS, hemorrhagic stroke; IS, ischemic stroke; IC, intracerebral; CAA, cerebral amyloid angiopathy

References	Design	Number of cases	Population	Diagnostic criteria	Conclusion
Tolppanen et al. (2013) [21]	Cohort study	50,808	Community-dwelling people of Finland	Prescription reimbursement register	AD patients have a higher chance of developing HS
Chi et al. (2013) [22]	Case-control matched analysis	980	AD cases	Database from Taiwan's National Health Insurance Research	Increased risk of IS and IC hemorrhage in AD cases
Woo et al. (2002) [25]	Population-based study	188	Hospitals in the Greater Cincinnati/Northern Kentucky region	Medical record and neuroimaging review	One-third of the patients of lobar IC hemorrhage possess an apolipoprotein E4 or E2 allele, and about half of all the cases of non-lobar IC hemorrhage are attributable to hypertension
Fekih-Mrissa et al. (2013) [27]	Case-control study	48	Tunisian population	Clinical diagnostic criteria of the National Institute of Neurological and Communicative Disorders and the Stroke and Alzheimer's disease and Related Disorders Association (NINCDSADRDA)	APO ɛ4 allele is associated with concomitant AD and stroke
Olichney et al. (1995) [28]	Retrospective clinic-pathological study	145	Autopsy series of the San Diego Alzheimer's Disease Research Center (ADRC)	Autopsy-confirmed cases of AD	Severe CAA and hypertension in AD patients produce multiplicative injuries to the neuronal vasculature and increase the frequency of cerebral infarction
Olichney et al. (2000) [29]	Review study	306	University of California, San Diego	The autopsy confirmed cases of AD	Advanced cases of CAA were associated with an increased risk of cerebrovascular events irrespective of APOE4 genotype
Khan et al. (2018) [30]	Systematic review	9027	People of European ancestry	Published and unpublished studies reporting on APOE genotype and IS	APOE genotype show a positive dose-response association with the IS

Clinical implications: Risk factors, epidemiological studies establishing the correlation

**Figure 3:**
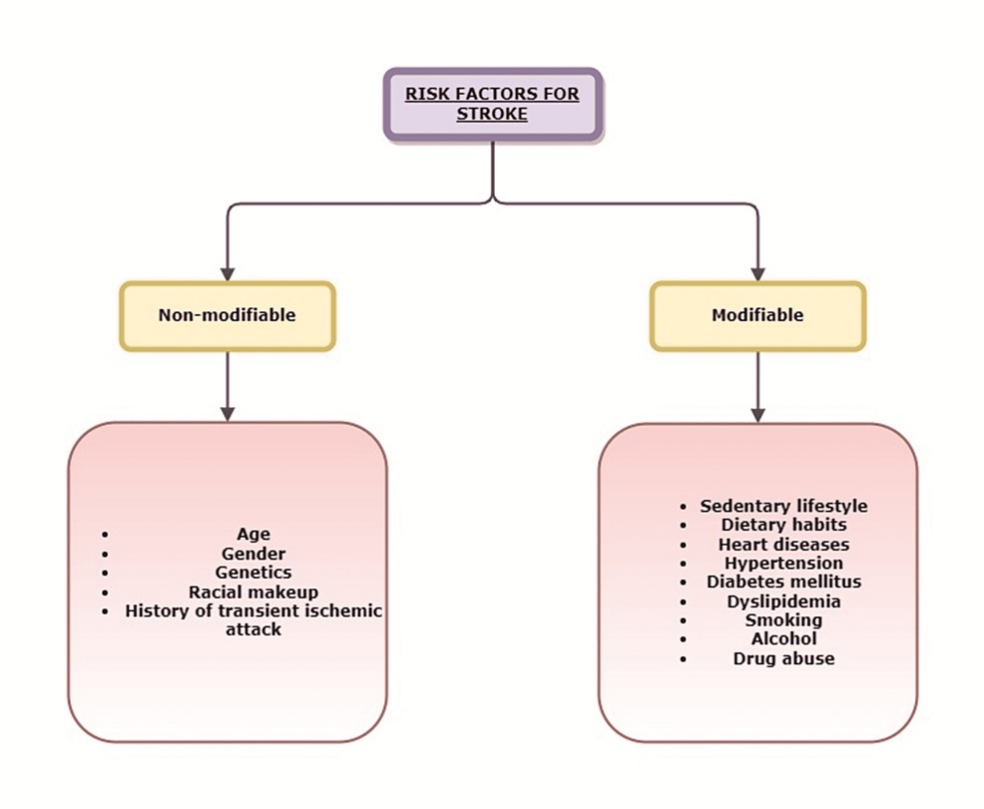
Risk factors for stroke Image credit: Anuradha Gupta

Risk factors for stroke include non-modifiable entities like old age, female gender, certain racial groups, hereditary makeup, and a previous history of transient ischemic attack [14]. Among modifiable risk factors, heart diseases, hypertension, diabetes mellitus, dyslipidemia, smoking, alcohol intake, drug abuse, diet, and sedentary lifestyle have a role in the majority of the cases (Figure 3) [14].

Patients with stroke typically present with sudden onset of neurological deficits such as facial, upper limb, or lower limb weakness, speech impairment, and visual field defects, or they may experience loss of consciousness or convulsions [31]. These presenting symptoms are per a prospective cohort study undertaken by Nor et al. in 2005 for one year considering 343 suspected stroke patients [31]. This study developed a Recognition of Stroke in the Emergency Room (ROSIER) scale comprising seven items with total scores ranging from -2 to +5 [31]. It was built based on clinical history (loss of consciousness, seizures) and neurological deficits (paralysis or paresis, aphasia, visual field defects) [31]. Suter et al. did a study on the brains of 184 autopsy cases (105 definite AD cases and 79 age-matched controls) in 2002 to know about the occurrence of watershed cortical infarcts in patients with AD [32]. After analysis by histochemical and immune-histochemical techniques, it was found that the degeneration and dementia in AD patients are aggravated, and worsened by white matter changes and cortical watershed micro-infarcts caused by cerebral hypoperfusion [32]. Prompt monitoring and treatment of blood pressure and arterial hypotension can be used to prevent these watershed cortical micro-infarcts [32].

Later in the year 2007, intending to know about the prognostic differences between AD and AD with the cerebrovascular disorder, Song et al. conducted a study in which 150 subjects participated, and the relation between concomitant silent cerebral infarctions and the cognitive decline of AD patients were studied and established [33]. After the study, it was clear that AD patients with the concomitant cerebrovascular disease have a more severe and early cognitive decline than those with no vascular pathology [33]. T2-weighted MRIs in AD patients frequently show the presence of small lesions in the form of black dots, which are micro-bleeds; the clinical relevance of these micro-bleeds is obscure [34]. It is suggested that such micro-bleeds are detrimental to cognition [34]. A paper published in 2012 by van der Flier WM suggested that these micro-bleeds pose an increased risk of stroke-related mortality in AD patients [34]. Another paper on micro-bleeds was published by Benedictus et al. in 2015 after performing a longitudinal cohort study on patients with AD within the memory clinic-based Amsterdam Dementia Cohort [35]. They deduced that non-lobar micro-bleeds in such patients increase the risk of cardiovascular events and mortality, whereas lobar micro-bleeds increase the risk for stroke and related mortality [35]. Prompt treatment with extreme care is needed in AD patients who possess micro-bleeds [35].

A systematic review was done by Rostamian et al. in 2014 using twelve prospective cohort studies comprising 82,899 subjects, of whom 3043 had an episode of incident stroke [36]. The aim was to establish the association between cognitive impairment and risk of stroke and know about the specific estimates of such association and identify the cognitive domains that associate most with incident stroke [36]. The result showed a clear-cut increased risk of stroke with prior cognitive impairment [36]. In the case of specific cognitive domains like memory, executive function, or language, this association showed no significant difference [36]. A retrospective cohort study published in 2015 by Cook et al. in the United Kingdom on 19,902 AD-dementia and matched non-AD-dementia patients concluded that AD-dementia is a risk factor for future occurrences of the HS [37]. This notion was further emphasized in a random-effects meta-analysis by Waziry et al. in 2020 using a relatively larger sample population from nationwide registries of Finland, Sweden, Taiwan, the United Kingdom, and the Netherlands [38]. They also assessed the rate of stroke occurrence per 1,000 person-years, which was 3.41 per 1000 person-years for HS among AD cases [38]. In light of the above studies, it appears that AD patients are at increased risk of both IS and HS. Also, cerebrovascular pathologies in AD patients lead to a more severe cognitive decline.

Any dementia preceding the onset of an index stroke in a patient is called preexisting dementia or pre-stroke dementia [39, 40]. A total of 9,304 patients presenting with an episode of acute IS between 2003 and 2008 using the Registry of the Canadian Stroke Network were enrolled in a retrospective cohort study by Saposnik et al. in 2011 [39]. Among these, 702 patients had preexisting dementia [39]. It was seen after the research that preexisting dementia imposes an increased burden on the health care system by increasing the rate of disability and institutionalization after an episode of acute IS [39]. In an attempt to unveil the association between pre-stroke dementia and functional outcome after stroke, Wakisaka et al. did a propensity score-matched cohort study in 2017 using 4,237 patients with IS registered in the Fukuoka Stroke Registry within 24 hours of onset, who were functionally independent before [40]. It was seen that the risk for neurological deterioration and poor functional outcome at three months after the onset of stroke in demented patients was significantly higher when compared with those without prior dementia [40]. After a year, Zupanic et al. did a retrospective cohort study using data in the Swedish national dementia registry (SveDem) and the Swedish national stroke registry (Riksstroke) [41]. They suggested that although patients with a diagnosis of dementia who suffer from an IS have equal access to direct stroke unit care in comparison to non-dementia patients, they receive lesser specific diagnostic tests and assessments in addition to having a shorter stay in a stroke unit and total hospitalization time [41]. In a cohort study done in Finland on community-dwellers with clinically diagnosed dementia in the year 2020, Tolppanen et al. concluded that during these patients' terminal care, it is vital to address cardio/cerebrovascular diseases because, in addition to neurological disorders, half of these patients die from cancers and cardio/cerebrovascular diseases [42].

A few researchers have suggested that people suffering from AD receive a suboptimal level of care [43]. To analyze this idea, Heiskanen et al. in 2015 performed an exposure-matched cohort study in Finnish community-dwellers with a clinical diagnosis of AD utilizing data on 30-day mortality after IS, HS, and myocardial infarction [43]. Even though the 30-day mortality in the AD cohort was slightly higher, it did not prove that the care these patients received was inferior to what is given to other patients [43]. The higher rate was due to the higher mortality of AD cases in general or to substandard care because of deficits in cognition in such patients [43]. Thus impaired cognition increases mortality in stroke patients with AD. Nearly 50% of patients with dementia and 30% of patients without dementia died one year after a stroke [44]. This data is derived from a national longitudinal cohort study done in 2021 by Zupanic et al. using 12,629 IS patients with dementia matched with 57,954 stroke patients without dementia as controls in the Swedish national registries which concluded that dementia is an independent predictor of death after IS [44].

Prevention strategies and future implications

About 10% of patients who suffer from an attack of stroke for the first time have dementia [45]. As a result, health professionals face specific challenges in the primary care and management of patients with dementia who are hospitalized for an acute episode of stroke [45]. The standard imaging modality for preliminary diagnosis of acute stroke is non-contrast computed tomography (CT) [46]. Frisoni et al., in the year 1995, concluded that in order to detect vascularity in patients of dementia, CT imaging is a necessary adjunct to historical and clinical data [47]. As per a prospective, multicenter study performed by Kidwell et al. in 2004 at Bethesda, MRI can be used as an alternative to CT in emergency stroke cases [46]. The above-mentioned studies [39], [40], [42], [44] and [46] are summarized in Table 3. In the year 2009, it was found by Kimberly et al. in a retrospective analysis using MRIs of 78 subjects with a diagnosis of CAA and 55 subjects with AD or mild defects in cognition that a relatively substantial proportion of living patients suffering from severe CAA had MRI evidence of small subacute infarcts which pose an increased burden of IC hemorrhages [48].

**Table 3:** Studies establishing the clinical implications regarding the occurrence of stroke in AD patients IS, Ischemic stroke; AD, Alzheimer's disease; SveDem, Swedish national dementia registry; Riksstroke, Swedish national stroke registry; MRI, magnetic resonance imaging; CT, computed tomography

References	Design	Sample size	Population	Conclusion
Saposnik et al. (2011) [39]	Retrospective cohort study	9304	Patients presenting with an acute IS in the Registry of the Canadian Stroke Network between 2003 and 2008	Preexisting dementia imposes an increased burden on the health care system by increasing the rate of disability and institutionalization after an acute IS episode
Wakisaka et al. (2017) [40]	Cohort study	4,237	Patients registered in the Fukuoka Stroke Registry from June 2007 to May 2015	The risk for neurological deterioration and poor functional outcome at three months after the onset of stroke was significantly higher in dementia patients
Tolppanen et al. (2020) [42]	Cohort study	Patients with AD=27,948 Patients without AD=27,948	Community-dwellers residing in Finland	In addition to diseases of the nervous system, about half of the patients with AD die due to cancers and cardio/cerebrovascular diseases
Zupanic et al. (2021) [44]	Longitudinal cohort study	Cases=12,629 Controls=57,954	Patients with dementia from Swedish national registries	After a stroke, dementia is an independent predictor of death
Kidwell et al. (2004) [46]	Prospective multicenter study	200	Patients from UCLA Medical Center and National Institutes of Health Stroke Center at Suburban Hospital, Bethesda	MRI can be used as a substitute for CT scans in emergency stroke cases

To conclude, a CT scan is the primary modality to diagnose stroke, whereas MRI can be used to diagnose both AD and stroke [11, 12, 46-48]. Scott et al. in the year 2018 conducted a cross-sectional observational study in which 340 community-dwelling elders underwent a clinical evaluation including brain MRI and neuropsychological tests, and revealed a significant prevalence of small vessel markers of cerebrovascular pathology such as small vessel infarcts and periventricular white matter hyper-intensity in individuals with AD diagnosis [49]. Furthermore, they concluded that it is necessary to appreciate how cerebrovascular pathology interacts with the neurodegenerative process of AD to serve as an additional target for therapeutic prevention and intervention in the upcoming years [49].

A significant goal of stroke therapy is to restore cerebral blood flow to minimize neuronal damage caused by ischemia and treat the resulting deficits in neurological function by using thrombolytic agents like tissue plasminogen activator, anticoagulation, antiplatelet pharmacotherapy, neuroprotective drugs, surgical modalities like carotid endarterectomy, and treatment of potential risk factors like hypertension, diabetes, hyperlipidemia, and alcohol and drug abuse [14, 15]. On the other hand, the mainstay of treatment for moderate to severe AD at present is AChEIs which increase the available acetylcholine within cholinergic synapses, and NMDA antagonist memantine which can hinder the cellular damage caused by the activation of NMDA receptors administered individually or in combination [16, 50, 51]. Nevertheless, these drugs do not affect the underlying pathophysiology of neurodegeneration in AD, which revolves around NPs and NFTs [52]. Currently, many researchers are attempting to evaluate various therapeutic targets like amyloid and tau pathology, neurochemicals, inflammatory pathways, mitochondria, neuroglial cells, and several lifestyle modifications [50]. Neuroprotective drugs which can counteract neuronal dysfunction and death to tackle the problem of neurodegeneration in AD are still under development [51]. Different processes like Aβ amyloid protein metabolism, inflammation, oxidation, hormonal cascade, and defects in adrenergic, serotonergic, glutamatergic, and dopaminergic pathways involved in stroke progression can potentially be targeted for any therapeutic, preventative interventions in future research [16]. Other advanced treatment options include anti-amyloid approaches (e.g., bapineuzumab, solanezumab, tarenflurbil, aducanumab, gantenerumab, crenezumab), secretase inhibitor (e.g., semagacestat, avagacestat), tyrosine kinase inhibitor (e.g., masitinib, nilotinib), glutamate receptor antagonist like riluzole, aggregation inhibitors, herbs like Ginkgo biloba, transition metal chelators (e.g., clioquinol), nonsteroidal anti-inflammatory drugs (e.g., indomethacin), antioxidants, lipid-lowering drugs like statins, anti-hypertensive agents, selective phosphodiesterase inhibitors, vitamins (E, B12, B6, folic acid), growth factors, hormones (e.g., estradiol) as well as agents that target neurotransmitter or neuropeptide alterations [4, 16, 52]. Therapeutic interventions that affect amyloid-related cascades starting early in the disease process may decrease the severity or even prevent dementia [6].

Lin et al. suggested after a retrospective analysis in 2016 on 37,352 dementia patients above the age of 50 years using Taiwan National Health Insurance Database from 1999 to 2008 that AChEIs use in them was associated with a lower risk of IS [53]. However, it did not increase their survival [53]. This finding can be compared along the lines of a cohort study by Tan et al. in 2018 on 44,288 people with dementia which concluded that using AChEIs in demented patients significantly reduces the risk of IS and death [54]. Using logistic regression analysis on 805 patients with dementia who were independent in basic activities of daily living before admission and registered in the Fukuoka Stroke Registry, Wakisaka et al. concluded in 2021 that the use of AChEIs before stroke reduces the risk for neurological deterioration and poor functional outcome [55]. Thus, AChEIs slow the progression of AD and can tackle the cerebrovascular component in the pathophysiology of AD.

Another drug, memantine, works by blocking the overstimulated NMDA receptors and preventing the neurotoxicity caused by the massive release of glutamate [56]. Few preclinical studies have shown that memantine decreases the volume of infarction in stroke patients and also improves neurological outcomes [56]. Thus it can be used as an adjunct and alternative agent to protect neuronal tissue, which has undergone ischemia [56]. So in a way, it can provide a window to minimize the brain damage until reperfusion therapy for stroke can be initiated [56]. Lipid-lowering drugs like statins have also shown efficacy in treating AD in a few cases by altering the regulation of amyloid-β and vascular function [16]. These drugs are also beneficial for IS. The double advantage of statins was tested in a longitudinal cohort study done by Petek et al. on 48,711 patients in Swedish registries in 2020 [57]. It was concluded that statins may benefit dementia patients in terms of survival and IS risk in a dose-dependent manner [57]. Aspirin is a common over-the-counter drug used by many older adults. The AD2000 Collaborative Group performed a randomized controlled trial in 2008 on 310 community-resident AD patients to assess the effects of aspirin on them [58]. It was found that after two years of treatment with open-label low-dose aspirin, patients with typical AD had no valuable benefit but an increased risk of fatal cerebral bleeds [58]. A systematic review and comparison by Thoonsen et al. in 2010 using two randomized controlled trials on aspirin for AD showed similar results [59]. It was demonstrated that although the use of aspirin for cardiovascular pathology does not affect cognition, its use in AD patients imposes an increased risk of IC hemorrhage [59]. Thus, people suffering from AD should avoid aspirin as it increases the chances of IC bleeds. A systematic review and meta-analysis done by Azarpazhooh et al. in 2018, using ten population-based cohort studies comprising 2856 subjects, suggested that cases of dementia can be prevented or slowed down if the vascular component of AD pathology is therapeutically targeted [60]. Studies focusing on therapeutic interventions that can benefit patients of AD who suffer from stroke are included in Table 4.

**Table 4:** Studies focusing on therapeutic interventions that can benefit patients of AD who suffer from stroke AChEIs, acetylcholinesterase inhibitors; IS, ischemic stroke; AD, Alzheimer's disease

References	Design	Sample size	Population	Conclusion
Lin et al. (2016) [53]	Retrospective analysis	37,352	Taiwan National Health Insurance Database from 1999 to 2008	AChEIs use in demented patients was associated with a lower risk of IS but no increase in survival
Tan et al. (2018) [54]	Cohort study	44,288	People with dementia registered in the Swedish Dementia Registry from 2007 to 2014	The use of AChEIs in dementia patients significantly reduces the risk of IS and death
Wakisaka et al. (2021) [55]	Logistic regression analysis	805	Patients with dementia registered in the Fukuoka Stroke Registry between June 2007 and May 2019	Treatment with AChEIs prior to stroke in AD patients significantly reduces the risk for future neurological deterioration and poor functional outcomes
Petek et al. (2020) [57]	Longitudinal cohort study	48,711	Patients in combined Swedish registries	Statins may benefit dementia patients in terms of survival and IS risk in a dose-dependent manner
AD2000 Collaborative Group (2018) [58]	Randomized controlled trial	310	Community-resident patients who had AD	After two years of treatment with open-label low-dose aspirin, typical AD patients had no valuable benefit but an increased risk of fatal cerebral bleeds
Azarpazhooh et al. (2018) [60]	Systematic review and meta-analysis	2856 subjects	Population-based cohort study before February 2017	Cases of dementia can be prevented or slowed down if the vascular component of AD pathology is therapeutically targeted

Although efforts to treat neurodegeneration associated with AD pathology have not been successful to date, it is fair to say that vascular risk factors associated with concurrent AD and stroke are very much preventable or even treatable [20]. For AD patients, prevention and treatment of vascular risk factors during their midlife should be implemented to reduce cognitive decline later in life [20].

Limitations

A discussion of specific therapies targeting stroke in patients with and without AD has not been included in this paper. Studies on the surgical interventions and their prognosis for stroke in AD patients have not been reviewed.

## Conclusions

The studies compiled in the presenting article show that AD and stroke are two interrelated entities. It is known that stroke is a risk factor for AD development. However, the data presented in the studies mentioned above suggest that certain aspects of AD pathophysiology can contribute to stroke development later in life. We also tried to unveil the linkage of the APOE genotype with the concomitant occurrence of AD and stroke. A few studies suggested that CAA cases are more likely to suffer from cerebrovascular events due to their effects on the BBB and the neuronal vasculature. This study tried to shed some light on the challenges that health professionals face when both diseases present simultaneously, and the deteriorating functional outcomes of cognitively impaired patients. We also spoke explicitly about the increased mortality caused by stroke in AD patients. AD, an irreversible neurodegenerative disorder, is not entirely treatable, but the cerebrovascular components of its pathophysiology are very much manageable if targeted with a proper therapeutic strategy. This article is instrumental in pointing out that an interdisciplinary approach to treatment starting early in AD patients with AChEIs and NMDA antagonists and an emphasis on minimization of cerebrovascular defects using appropriate drug therapy have a considerable benefit in the progression or even prevention of stroke later in life. Such an approach in the management of AD can be pivotal in improving the quality of life and standard of living in people suffering from cognitive impairment and reducing the burden of care in the health care setting. Intensive studies aiming at acquiring knowledge about the factors making AD a contributor to stroke should be further explored with a larger number of subjects. Further emphasis on more in-depth research on better management and therapeutic intervention of AD patients targeting their cerebrovascular pathology, in particular, is a need for the future.
